# Does local steroid therapy improve outcomes in acute otitis media with serous labyrinthitis? A retrospective analysis

**DOI:** 10.1007/s00405-026-10616-y

**Published:** 2026-10-20

**Authors:** H. T. Fischer, F. Keller, C. Schmit, F. Theurl, M. Santer, A. Hils, L. Stecher, F. Koller, L. Schmutzler, T. Gottfried, J. Schmutzhard, B. Hofauer, D. Dejaco

**Affiliations:** 1 Universitätsklinik Für Hals, Nasen- Und Ohrenheilkunde, Medizinische Universität Innsbruck, Anichstraße 35, A-6020 Innsbruck, Österreich; 2 Univ.-Klinik Für Innere Medizin IV (Nephrologie Und Hypertensiologie), Medizinische Universität Innsbruck, Innsbruck, Österreich

**Keywords:** Acute otitis media, Sensorineural hearing loss, Serous labyrinthitis, Intratympanic glucocorticoids, Corticosteroid therapy, Inner ear involvement, Bone conduction puretone average, Middle ear drainage, Ventilation tube insertion, Retrospective study

## Abstract

**Purpose:**

Sensorineural hearing loss represents a rare but clinically relevant complication of acute otitis media (AOM). Although intratympanic glucocorticoid (GC) therapy is established in other cochleovestibular disorders, its efficacy in this context remains insufficiently investigated. The aim of this study was to retrospectively assess whether local intratympanic glucocorticoid application is associated with improved clinical outcomes in patients with acute otitis media–related sensorineural hearing loss.

**Methods:**

A retrospective analysis was performed on patients diagnosed with AOM-associated inner ear involvement between January 2014 and November 2024. Patients were stratified according to GC treatment regimen: (1) intratympanic application of glucocorticoids (IC), (2) no intratympanic application of glucocorticoids (NIC). Subgroup analyses were performed according to the surgical drainage method and severity of inner ear involvement. Primary endpoint was improvement in bone-conduction pure-tone average (BCPTA4).

**Results:**

A total of 137 patients were included (IC: 44, NIC: 93). Sex, affected side and subjective improvement did not differ significantly between both groups (all *p* > 0.08). Both groups demonstrated significant hearing improvement following treatment (*p* < 0.001). Although numerical trends favored the NIC group across different severity levels, no statistically significant differences in BCPTA4 improvement were observed between treatment modalities (*p* = 0.8); however, the non-randomized design and baseline group differences limit interpretation. In exploratory analyses, Ventilation Tube Insertion (VT) and CO₂-laser myringotomy (LP) were associated with significant hearing recovery (*p* < 0.001).

**Conclusions:**

Both treatment regimens (IC, NIC) led to significant hearing improvement in AOM-associated inner ear involvement. In this non-randomized comparison, no statistically significant difference in BCPTA4 improvement was detected between groups; however, selection bias, baseline imbalances and residual confounding may have obscured small treatment effects. These findings do not constitute evidence of equivalence.

**Supplementary Information:**

The online version contains supplementary material available at https://doi.org/10.1007/s00405-026-10616-y.

## Introduction

Acute otitis media (AOM) is a frequently encountered condition in clinical practice. Although complications of AOM have become increasingly rare due to the widespread early administration of antibiotics, they continue to pose serious complications that usually require immediate and adequate treatment [1–3]. In addition to acute mastoiditis, known complications include facial nerve palsies and potentially life-threatening intracranial complications [3–5].

Another clinically relevant complication to consider is serous labyrinthitis, caused by diffusion of inflammatory toxins into the inner ear, leading to AOM-associated inner ear involvement [6–9]. In this study, “AOM-associated inner ear involvement” (used synonymously with serous labyrinthitis) is operationally defined as unilateral AOM with audiometrically confirmed sensorineural hearing loss (elevated bone-conduction thresholds) in the absence of alternative etiologies. The diagnosis was based on clinical and audiometric criteria; imaging confirmation was not required. Treatment of otitis media with sensorineural hearing loss includes symptomatic and antibiotic treatment as well as drainage of the inflammatory fluid from the middle ear. In addition to the usual paracentesis, the regeneration of the toxically damaged hair cells is supported by the administration of anti-inflammatory glucocorticoids [8].

Anti-inflammatory glucocorticoids can be administered systemically or locally via intratympanic cortisone injection. The intratympanic administration of glucocorticoids has become increasingly established in the treatment of idiopathic sudden sensorineural hearing loss due to the absence of systemic side effects and the higher perilymph levels demonstrated in animal models [10–12]. Despite their widespread use, there have been few studies investigating the potential benefit of intratympanic cortisone injections in the treatment of AOM with sensorineural hearing loss [13].

The aim of the present study is to retrospectively investigate whether intratympanic application of glucocorticoids can significantly improve the therapeutic outcome in patients with sensorineural hearing loss associated with AOM.

## Materials and methods

**Fig. 1:**
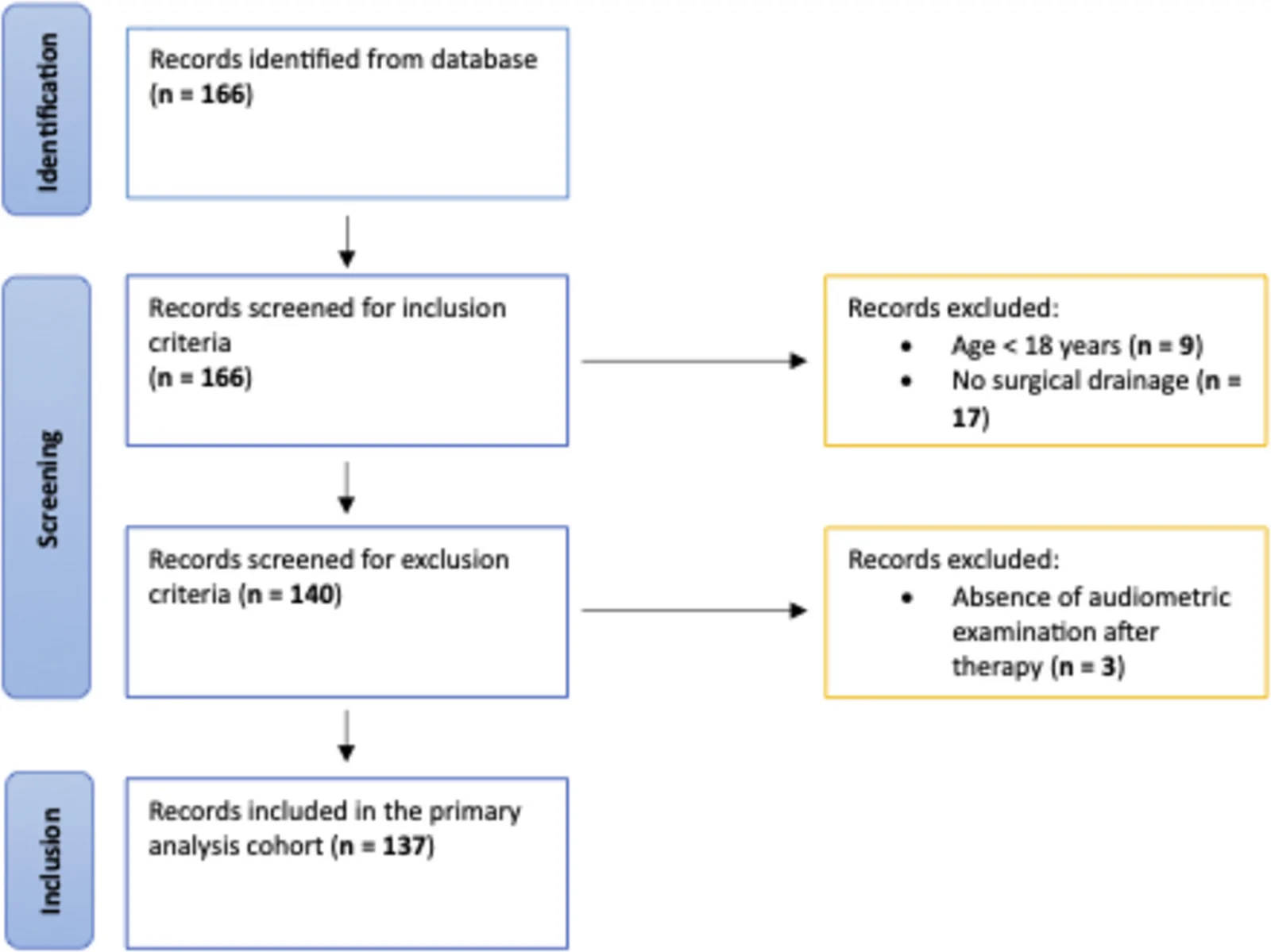
Flow chart of patient selection and formation of the primary analysis cohort

We retrospectively analyzed the medical records of adult patients diagnosed with AOM with associated sensorineural hearing loss, who were admitted to the Department of Otorhinolaryngology, Head and Neck Surgery at the Medical University **XXXXX** between January 2014 and November 2024. Audiometric records were available for 166 patients. Of these, 29 did not meet the inclusion criteria or met one or more exclusion criteria, resulting in a primary analysis cohort of 137 patients (NIC: 93; IC: 44) (Fig. 1). The study adhered to the principles outlined in the Declaration of Helsinki and Good Clinical Practice guidelines.

### Inclusion criteria

The inclusion criteria were:

- Diagnosis of unilateral AOM with audiometrically confirmed inner ear involvement
- Initiation of treatment within 14 days of symptom onset
- Therapy involves surgical drainage of the middle ear
- Age ≥ 18 years

Inner-ear involvement was defined as an elevated bone-conduction pure-tone Average (BCPTA4; 500 Hz, 1, 2, and 4 kHz) confirmed by audiometry and accompanied by clinical suspicion of serous labyrinthitis. Audiometry was routinely performed as part of the inpatient diagnostic work-up.

### Exclusion criteria

The exclusion criteria were:

- Determined deviating etiology of unilateral sensorineural hearing loss
- Discontinuation of the therapy regimen
- Absence of audiometric examination after completion of therapy

### Study population

Patients were managed according to institutional practice for acute otitis media with suspected inner-ear involvement, typically comprising systemic antibiotic therapy and a short course of systemic glucocorticoids. Systemic glucocorticoids were documented in 122 of 137 patients (89%; Table 1); residual confounding by systemic therapy cannot be excluded. The antibiotic regimen, including the choice of agent, route of administration, and treatment duration, was tailored to the individual patient's clinical presentation and treating physician's assessment.

**Table 1:** Clinical characteristics and outcome parameters in the entire cohort and by treatment group

*Characteristic*	Overall (*N* = 137)^1^	NIC-group (*N* = 93)^1^	IC-group (*N* = 44)^1^	*p*-value^2^
*Sex*				0.850
*Male*	49 (36%)	34 (37%)	15 (34%)	
*Female*	86 (64%)	59 (63%)	29 (66%)	
*Side*				0.716
*Right*	75 (55%)	52 (56%)	23 (52%)	
*Left*	62 (45%)	41 (44%)	21 (48%)	
*Age*	46 (34, 59)	43 (32, 53)	56 (37, 67)	0.002
*Pretreatment*				< 0.001
*None*	15 (11%)	5 (5%)	10 (23%)	
*Syst. GC*	122 (89%)	88 (95%)	34 (76%)	
*Preop. BCPTA4 (dB) *^*1*^	29 (21, 41)	29 (20, 35)	39 (24, 50)	0.002
*Tercile subgroups*				< 0.001
* 1 st subgroup*	46 (34%)	35 (38%)	11 (25%)	
*2nd subgroup*	45 (33%)	37 (40%)	8 (18%)	
*3rd subgroup*	46 (34%)	21 (23%)	25 (57%)	
*Surgical method*				0.007
*PZ*	8 (6%)	6 (75%)	2 (25%)	
*VT*	96 (70%)	72 (75%)	24 (25%)	
*LP*	33 (24%)	15 (45%)	18 (45%)	
*Subj. Improvement*	89 (65%)	65 (70%)	24 (55%)	0.088
Postop. BCPTA4 (dB)				
First	25 (19, 36)	24 (18, 33)	32 (20, 51)	0.015
Final	21 (13, 31)	19 (13, 25)	24 (14, 43)	0.012
*Change in BCPTA4*				
*Absolute (dB)*	−8 (−13, −1)	−8 (−13, −1)	−6 (−16, −1)	0.754
*Relative (%)*	−27 (−48, −5)	−30 (−48, −7)	−19 (−50, −3)	0.325
*Complications*	13 (10%)	9 (10%)	4 (9%)	

^1^Median (Q1, Q3) ^2 ^Wilcoxon rank sum test; Fisher’s exact test

The study population consisted of two treatment groups based on the treatment modality. The following groups were defined in our retrospective analysis:

*Non-intratympanic glucocorticoid group (NIC)*

Patients in this group underwent surgical drainage of the middle ear with or without additional systemic glucocorticoid therapy. Systemic treatment, when administered, consisted of high-dose glucocorticoids over a consecutive period of three days, given either enterally or parenterally. No local (intratympanic) glucocorticoid application was performed in this group. Patients were subsequently monitored in an outpatient setting.

*Intratympanic application of glucocorticoids (IC)*

In addition to surgical drainage of the middle ear, patients in this group underwent a targeted glucocorticoid application (Dexabene® 4 mg) into the middle ear under general anesthesia as part of the surgical intervention. Subsequent glucocorticoid applications were performed without anesthesia. The treatment regimen consisted of repeated intratympanic glucocorticoid administration over three consecutive days, with or without additional systemic high-dose glucocorticoid therapy. Patients were advised on post-application care and monitored accordingly.

Intratympanic glucocorticoid therapy was not assigned according to a predefined protocol but at the discretion of the treating physician. Treatment allocation was based on clinical judgment and could be influenced by patient age, the severity of inner-ear involvement, and surgeon preference.

A further subdivision into three subgroups was carried out based on the surgical method used for drainage of the middle ear. These surgical-modality comparisons were prespecified as exploratory subgroup analyses.

The choice of middle ear drainage procedure was not protocolized and was made at the treating surgeon’s discretion according to the clinical presentation. In institutional practice, ventilation tubes were typically considered when ongoing middle-ear ventilation was required; CO₂-laser myringotomy for short-term controlled drainage in selected acute cases; and simple myringotomy for brief drainage only. These modality comparisons remain exploratory.

*Myringotomy without Ventilation Tube Insertion (PZ)*

Patients in this group underwent myringotomy under local anesthesia. Depending on the study group, the procedure was supplemented by the intratympanic application of glucocorticoids (Dexabene® 4 mg). Subsequent glucocorticoid applications were performed in local anesthesia. Patients received instructions on post-application care and were monitored for an appropriate period.

*Myringotomy with Ventilation Tube Insertion (VT)*

Patients in this group underwent myringotomy with subsequent insertion of a ventilation tube under general anesthesia. Depending on the study group, the procedure was supplemented by the intratympanic application of glucocorticoids (Dexabene® 4 mg). Subsequent glucocorticoid applications were performed without anesthesia. Patients received instructions on post-application care and were monitored for an appropriate period.

*Myringotomy with CO₂-laser (LP).*

Patients in this group underwent myringotomy with CO₂ laser under general anesthesia. Depending on the study group, the procedure was supplemented by the intratympanic application of glucocorticoids (Dexabene® 4 mg). Subsequent glucocorticoid applications were performed without anesthesia. Patients received instructions on post-application care and were monitored for an appropriate period.

### Audiometry examination

Audiometry was conducted by a trained audiologist. The bone-conduction pure-tone Average (BCPTA4) was calculated using hearing threshold at 500 Hz, 1 kHz, 2 kHz and 4 kHz. The resulting BCPTA4 were further subdivided into tercile subgroups of the calculated pre-therapeutic hearing threshold, consisting of the same number of patients. Baseline audiometric findings are shown in Supplementary Table 2.

### Assessment of outcome

The primary outcome measure was post-therapeutic hearing improvement and time to improvement.

*Absolute Hearing improvement*

Pre-, and post-therapeutic BCPTA4 were calculated after therapy completion. Postoperative BCPTA4 was collected at several different time points.

Hearing improvement was defined as the difference between baseline BCPTA4, obtained at the time of initial presentation prior to intervention, and the final post-therapeutic BCPTA4 within a follow-up period of up to 10 weeks. In the primary analysis set, the median interval from baseline to outcome audiogram was 7 days (IQR 3–22) overall; 6 days (IQR 3–21) in NIC and 12.5 days (IQR 3.8–26) in IC (*p* = 0.223).

*Time to improvement (TTI)*

The time to relevant improvement of the hearing threshold measured post-therapeutically was recorded. A validated minimal clinically important difference (MCID) for BCPTA4 in AOM-associated sensorineural hearing loss has not been established; the ≥10 dB threshold was chosen pragmatically based on clinical convention.

*Subjective improvement*

During follow-up examinations, patients were asked to subjectively assess their hearing improvement. Subjective improvement was documented for each patient.

*Complications*

The patient files were examined for possible follow-up interventions caused by the therapeutic intervention. Any necessary operative revision was documented.

### Statistical analysis

Descriptive statistics are presented as median and interquartile range (IQR) for continuous variables and as absolute and relative frequencies for categorical variables.

Unless stated otherwise, analyses of the primary bone-conduction endpoint were performed in the subset of patients with a non-missing anchored BC pair, defined as baseline BCPTA4 (earliest bone-conduction audiogram from first presentation through the day of intravenous therapy, inclusive) and outcome BCPTA4 (latest bone-conduction audiogram strictly after the intravenous therapy day, with test date no later than 70 calendar days after the baseline test date). Absolute hearing improvement (ΔBCPTA4) was defined as outcome minus baseline BCPTA4; negative values indicate improvement.

Between-group comparisons across the two treatment groups (NIC vs. IC) were performed using the Wilcoxon rank-sum test for continuous variables and Fisher’s exact test for categorical variables. The primary comparison for ΔBCPTA4 was based on this non-parametric framework. Within-group changes from baseline to outcome were assessed using the Wilcoxon signed-rank test; paired effect sizes (Cohen’s d) are reported where appropriate.

Comparisons across more than two groups (baseline BCPTA4 tertiles and surgical drainage modality) were conducted using the Kruskal–Wallis rank-sum test for continuous variables and Fisher’s exact test for categorical variables. Baseline BCPTA4 tertiles were defined as equally sized groups based on the rank distribution.

Time to clinically relevant hearing improvement was defined as the time from baseline to the first ≥ 10 dB improvement in BCPTA4, assessed on bone-conduction audiograms strictly after the intravenous therapy day. Follow-up was administratively censored at 70 days after the baseline test date. Time-to-event analyses were performed using Kaplan–Meier methods, with group comparisons based on the log-rank test and Cox proportional hazards regression, reporting hazard ratios (HRs) with 95% confidence intervals (CIs).

To address confounding due to the non-randomized study design, inverse probability of treatment weighting (IPTW) targeting the average treatment effect (ATE) was applied as a prespecified sensitivity analysis. Propensity scores were estimated using logistic regression including baseline covariates (demographics, baseline hearing thresholds, treatment characteristics, and surgical modality). IPT-weighted Cox regression models with robust standard errors were used for time-to-event analyses, and weighted Kaplan–Meier curves were generated. In addition, supplementary analyses report mean differences in ΔBCPTA4 based on unweighted and IPT-weighted linear models; corresponding p values are considered descriptive and do not replace inference from the primary non-parametric analysis.

Covariate balance after weighting was assessed using standardized mean differences (SMDs). Following IPTW, absolute SMDs were < 0.15 for most covariates, including age and baseline BCPTA4. Residual imbalance persisted only for the infrequent PZ indicator (Supplementary Fig. 3).

All hypothesis tests were two-sided, and a *p*-value < 0.05 was considered statistically significant. Analyses were performed using R (Version 4.5.2).

## Results

### Clinical characteristics of the entire study population

As displayed in Table 1, the study included a total of 137 patients, of whom 64% were female and 36% were male, resulting in a female-to-male ratio of 1.8:1. AOM with associated sensorineural hearing loss affected the right ear in 55% and the left ear in 45%. The median age of the study cohort was 46 years. The median interval between clinical presentation and respective intervention was 1 day.

In addition to surgical drainage of the middle ear, systemic application of glucocorticoids was performed in 89%, while in 11% no systemic treatment with glucocorticoids was given. Regarding the treatment groups, 68% received no additional intratympanic application of glucocorticoids (NIC-group) while in 32% patients underwent an intratympanic glucocorticoid application (IC-group).

Regarding the surgical methods used in drainage of the middle ear, treatment modalities, 6% underwent myringotomy (PZ), 24% had laser paracentesis (LP) and 70% underwent ventilation tube insertion (VT).

The median preoperative BCPTA4 for the entire cohort was 29 dB. The NIC group had a median preoperative BCPTA4 of 29 dB, while the IC group had a median preoperative BCPTA4 of 39 dB.

### Clinical characteristics of the subgroups

The main study cohort was further divided into terciles based on the distribution of the pre-therapeutic hearing thresholds, i.e. the 33rd and 66th percentiles were used as cut-off values. Descriptive statistics are displayed in Table 2.

* 1 st Subgroup BCPTA4 Range (5 - 23.8 dB)*

The first subgroup comprises patients with a preoperative BCPTA4 at or below the 33rd percentile of the pretherapeutic hearing threshold distribution, ranging from 5 to 23.8 dB.

The median preoperative BCPTA4 value for the first subgroup was 19 dB.

The first subgroup consists of 46 patients of which 63% were female, while 37% were male. In 59% the right side was affected, while the left side was impacted in 41%. The median age of the first subgroup was 36 years.

76% of patients received only systemic glucocorticoid therapy in addition to surgical drainage of the middle ear, while in 24% intratympanic glucocorticoid application was used in addition to the treatment regimen just mentioned.

In the first subgroup 7% received drainage by myringotomy (PZ), 72% underwent tube insertion (VT), and 22% had laser paracentesis (LP).

*2*^*nd*^* Subgroup BCPTA4 Range (23.8 −36.2 dB)*

The second subgroup consists of patients with a preoperative BCPTA4 between the 34^th^ and the 66^th^ percentile of the pretherapeutic hearing threshold distribution, ranging from 23.8 to 36.2 dB.

The median preoperative BCPTA4 value for the second subgroup was 29 dB.

The subgroup consists of 45 patients of which 58% were female, while 42% were male. In 38% the right side was affected, while the left side was impacted in 62%. The median age of the second subgroup was 43 years.

82% of patients received only systemic glucocorticoid therapy in addition to surgical drainage of the middle ear, while in 18% intratympanic glucocorticoid application was used in addition to the treatment regimen just mentioned.

In the second subgroup 9% received drainage by myringotomy (PZ), 73% underwent tube insertion (VT), and 18% had laser paracentesis (LP).

*3*^*rd*^* Subgroup BCPTA4 Range (37.5, 75 dB)*

The third subgroup includes patients with a preoperative BCPTA4 above the 66^th^ percentile of the pretherapeutic hearing threshold distribution, ranging from 37.5 to 75 dB.

The median preoperative BCPTA4 value for the third subgroup was 46 dB.

The subgroup consists of 46 patients of which 72% were female, while 28% were male. In 67% the right side was affected, while the left side was impacted in 33%. The median age of the third subgroup was 60 years.

46% of patients received only systemic glucocorticoid therapy in addition to surgical drainage of the middle ear, while in 54% intratympanic glucocorticoid application was used in addition to the treatment regimen just mentioned.

In the third subgroup 2% received drainage by myringotomy (PZ), 65% underwent tube insertion (VT), and 33% had laser paracentesis (LP).

**Table 2:** Outcome parameters in the equally distributed severity tercile subgroups

*Characteristic*	Overall (*N* = 137)	1 st subgroup (*N* = 46)	2nd subgroup (*N* = 45)	3rd subgroup (*N* = 46)	*p*- value
*Sex*					0.373
*Male*	49 (36%)	17 (37%)	19 (42%)	13 (28%)	
*Female*	88 (64%)	29 (63%)	26 (58%)	33 (72%)	
*Side*					0.014
*Right*	75 (55%)	27 (59%)	17 (38%)	31 (67%)	
*Left*	62 (45%)	19 (41%)	28 (62%)	15 (33%)	
*Age*	46 (34, 59)	36 (29, 47)	43 (34, 53)	60 (49, 70)	< 0.001
*Pretreatment*					0.016
*None*	15 (11%)	2 (4%)	3 (7%)	10 (22%)	
*Syst. GC*	122 (89%)	44 (96%)	42 (93%)	36 (78%)	
*Preop. BCPTA4 (dB)*	29 (21, 41)	19 (14, 21)	29 (28, 33)	46 (41,55)	< 0.001
*Treatment group*					
*NIC*	93 (68%)	35 (76%)	37 (82%)	21 (46%)	
*IC*	44 (32%)	11 (24%)	8 (18%)	25 (54%)	
*Surgical method*					0.372
*PZ*	8 (6%)	3 (7%)	4 (9%)	1 (2%)	
*VT*	96 (70%)	33 (72%)	33 (73%)	30 (65%)	
*LP*	33 (24%)	10 (21%)	8 (18%)	15 (33%)	
*Subj. Improvement*	89 (65%)	31 (67%)	30 (67%)	28 (61%)	0.811
Postop. BCPTA4 (dB)					
First	25 (19, 36)	16 (13, 20)	26 (23, 31)	39 (35, 53)	< 0.001
Final	21 (13, 31)	11 (8, 18)	21 (18, 25)	36 (24, 45)	< 0.001
*Change in BCPTA4*^*1*^					
*Absolute (dB)*	−8 (−13, −1)	−4 (−10, 0)	−9 (−14, −3)	−9 (−20, −3)	0.008
*Relative (%)*	−27 (−48, −5)	−29 (−53, 0)	−29 (−44, −8)	−20 (−48, −5)	0.917
*Complications*	13 (10%)	4 (9%)	5 (11%)	4 (9%)	0.875

^1^Median (Q1, Q3) ^2^Kruskal-Wallis rank sum test; Fisher’s exact test

### Assessment of outcome in the entire study population

Of the 137 patients included a total of 65% reported a subjective improvement in hearing after treatment. The subjective improvement was higher in the NIC group (70%) than in the IC group (55%). No significant difference in subjective improvement was found depending on surgical drainage method (*p* = 0.284).

Complications were reported in 10% of cases in the studied cohort. Superinfection and dislocation of the placed ventilation tube were among the most frequently reported complications at 36% and 21% respectively. Removal of the dislocated ventilation tube was performed in 21% of the reported complications, while surgical revision with tympanoplasty was indicated in 2 cases.

No significant difference in complication rate was found between NIC group (9.7%) and IC group (9.1%). At 13% complications were highest in the VT subgroup, while no complications occurred in the LP and the PZ subgroups.

The median postoperative BCPTA4 in the entire study population was 25 dB for BCPTA4^first^ and 21 dB for BCPTA4^final^, both representing a significant improvement over preoperative BCPTA4 (*p* < 0.001). The NIC group had a median postoperative BCPTA4^first^ of 24 dB and a median postoperative BCPTA4^final^ of 19 dB. In the IC group, the BCPTA4^first^ measured at the first postoperative time point was 32 dB, while the median BCPTA4^final^ was 24 dB. The median absolute improvement in hearing threshold (preBCPTA4-postBCPTA4^final^) achieved by the respective therapy was −8 dB in the entire study collective. In the NIC group a median improvement of −8 dB was achieved, while the IC group showed a median improvement in hearing threshold of −6 dB (*p* = 0.754).

In the subgroups median absolute improvement in hearing threshold (preBCPTA4-postBCPTA4^final^) was measured at −5 dB in the PZ subgroup, −4 dB in the LP subgroup and −8 dB in the VT subgroup (*p* = 0.270).

The time to improvement among patients who demonstrated hearing recovery was 7 days in the overall cohort. Stratified by treatment group, the median time to improvement was 5.5 days in the NIC group and 9.0 days in the IC group. In subgroup analyses, the median time to improvement among improvers was 15.0 days in the PZ subgroup, 14.5 days in the LP subgroup, and 3.5 days in the VT subgroup.

### Assessment of outcome in the tercile subgroups

The main study population was further divided into three equally distributed subgroups based on the calculated pre-therapeutic BCPTA4.

*1*^*st*^* Subgroup*

Of the 46 patients, 67% reported subjective improvement in hearing during therapy. Median postoperative BCPTA4^final^ for the first subgroup was 11 dB. The median absolute hearing threshold improvement in the first subgroup was −4 dB while the median relative hearing threshold improvement was −29%.

*2*^*nd*^* Subgroup*

In the second subgroup (45 patients) a total of 67% reported subjective improvement in hearing during therapy. Median postoperative BCPTA4 for the second subgroup was 21 dB. The median absolute hearing threshold improvement in the second subgroup was −9 dB while the median relative hearing threshold improvement was −29%.

*3*^*rd*^* Subgroup*

Of the 46 patients, 61% reported subjective improvement in hearing during therapy. Median postoperative BCPTA4 for the third subgroup was 36 dB. The median absolute hearing threshold improvement in the third subgroup was −9 dB while the median relative hearing threshold improvement was −20%.

### Statistical evaluation of the main cohort

No significant differences between the two therapy modality groups in terms of sex and affected side (both *p* > 0.7) have been noticed. Subjective improvement and encountered complications also did not differ significantly between both groups (*p* = 0.088 and *p* = 1.0 respectively). Age and type of pretreatment differed significantly between NIC and IC groups (*p* = 0.002 and *p* < 0.001 respectively).

**Fig. 2:**
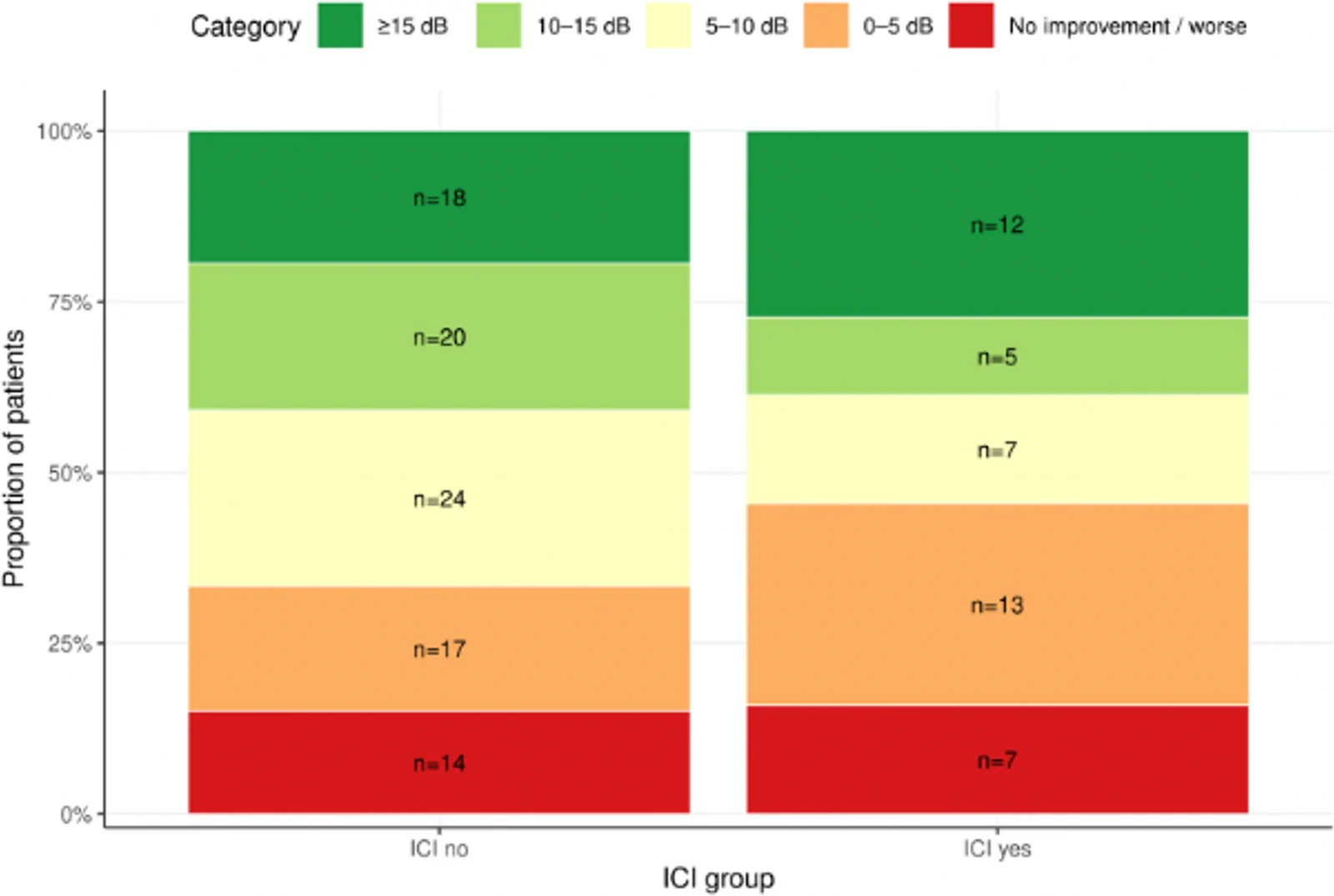

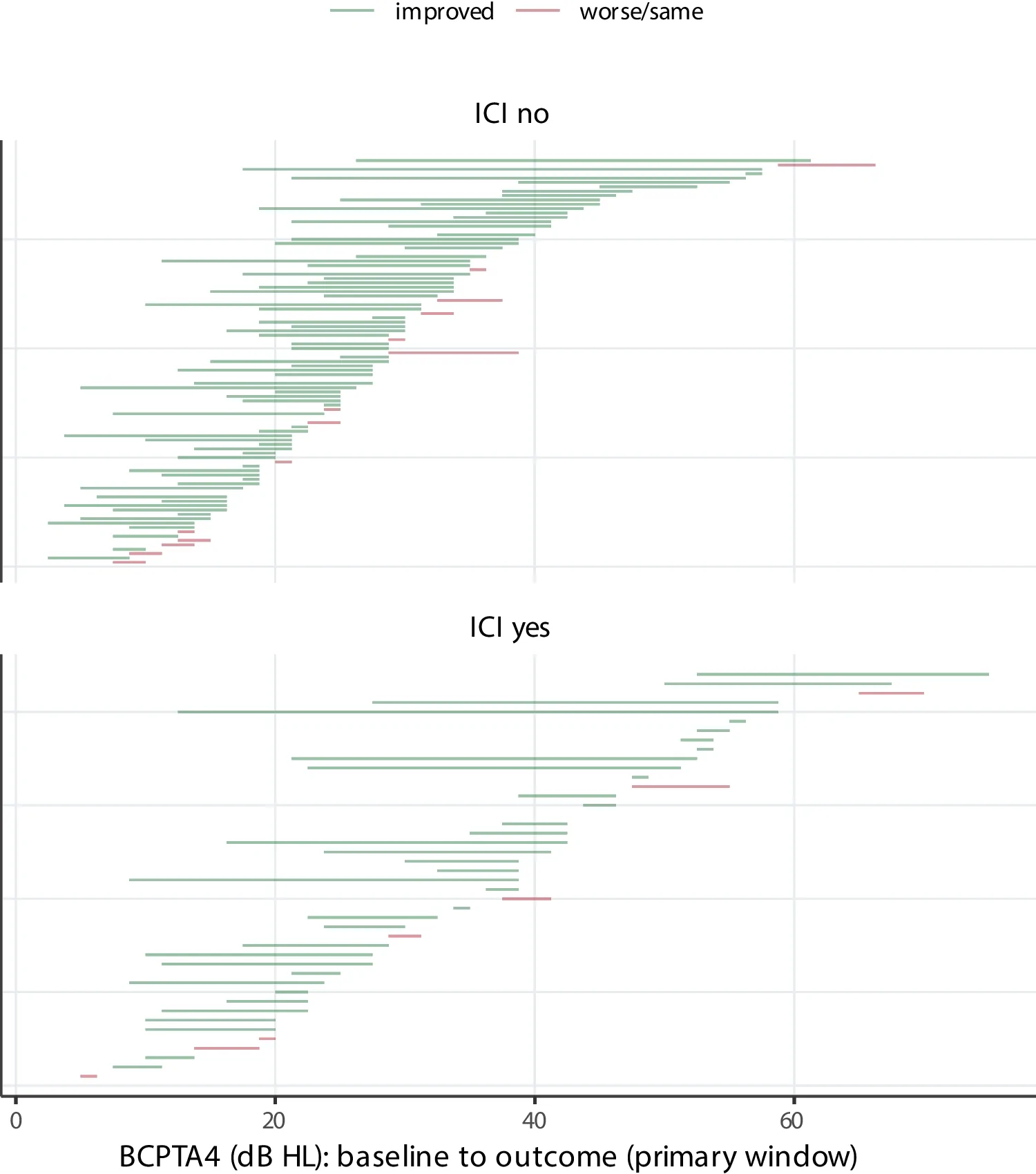
Change of BCPTA4 by treatment modality. **a**) Stacked distribution of BCPTA4 improvement categories by ICI group. **b**) Individual BCPTA4 from baseline to outcome; one segment per patient. Green: improvement; red: no improvement or worse

Preoperative and postoperative BCPTA4 differed significantly between both therapy modality groups (*p* = 0.002 and *p* = 0.012 respectively). In both groups a significant change in hearing threshold with large and medium effect size was observed post-therapy (NIC: *p* < 0.001; Cohen´s d = 0.94 and IC: *p* < 0.001; Cohen´s d = 0.79). No significant differences in the relative or absolute change in BCPTA4 were observed between the groups (*p* = 0.325 and *p* = 0.754 respectively) (Fig. 2).

**Fig. 3:**
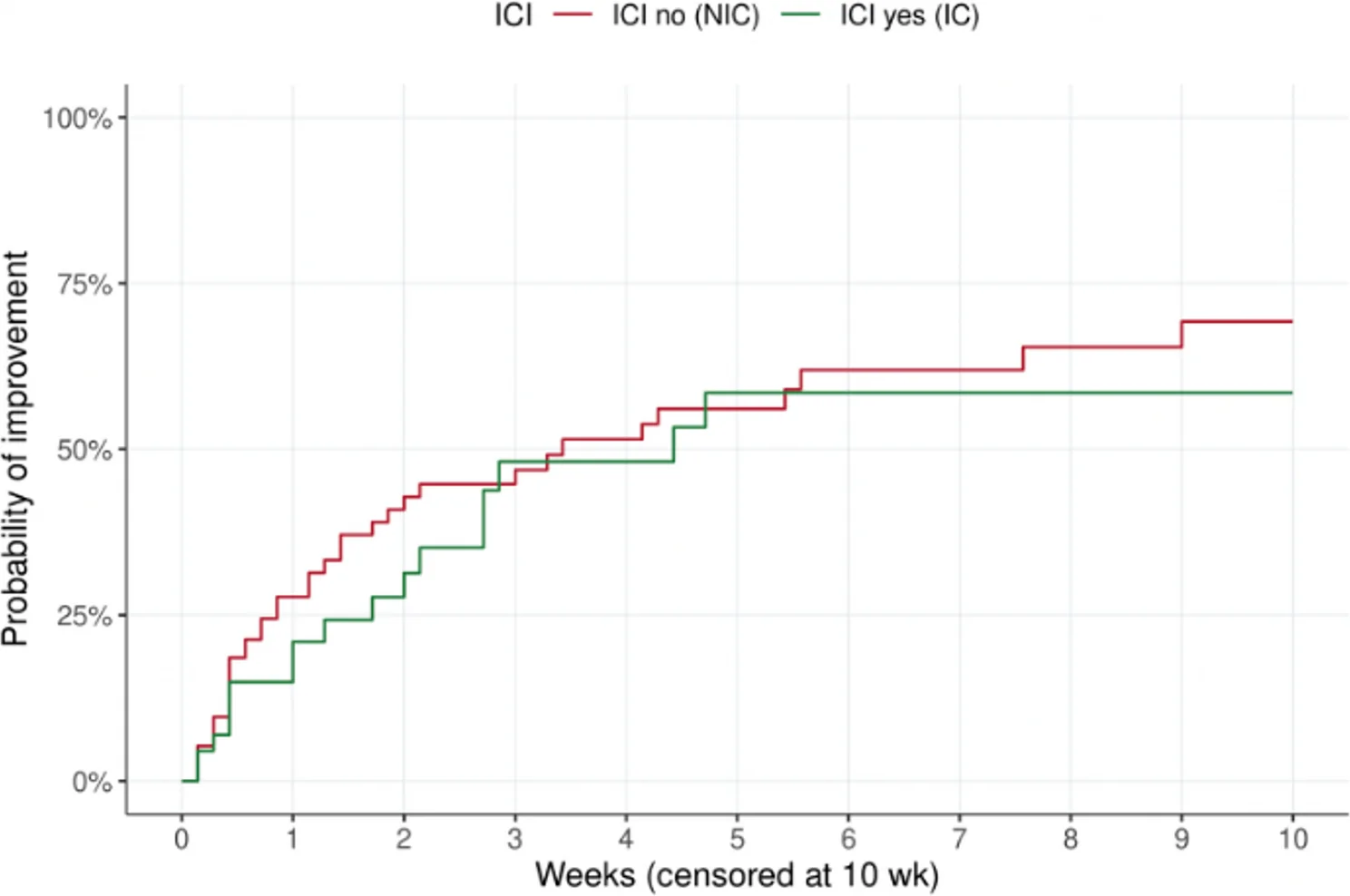
Time to improvement probability by treatment modality. Kaplan–Meier cumulative incidence of ≥ 10 dB BCPTA4 improvement (unweighted)

Regarding the time to improvement of the hearing threshold measured posttherapeutically, the Unweighted Cox regression showed no significant difference between treatment groups (HR = 0.78, 95% CI 0.44–1.37, *p* = 0.38) (Fig. 3). Findings remained non-significant after IPT-weighting (HR = 1.10, 95% CI 0.66–1.84, *p* = 0.73) (Supplementary Fig. 1).

### Statistical evaluation of the subgroups

In accordance with the subdivision made, a significant difference in pre-, and posttherapeutic BCPTA4 between the tercile subgroups (both *p* < 0.001) was found. Additionally, there were significant differences between all three subgroups in regard to age (*p* < 0.001), affected side (*p* = 0.014) and therapy modality (*p* < 0.001). Subjective improvement and occurred complications did not differ significantly between subgroups (all *p* > 0.8).

**Fig. 4:**
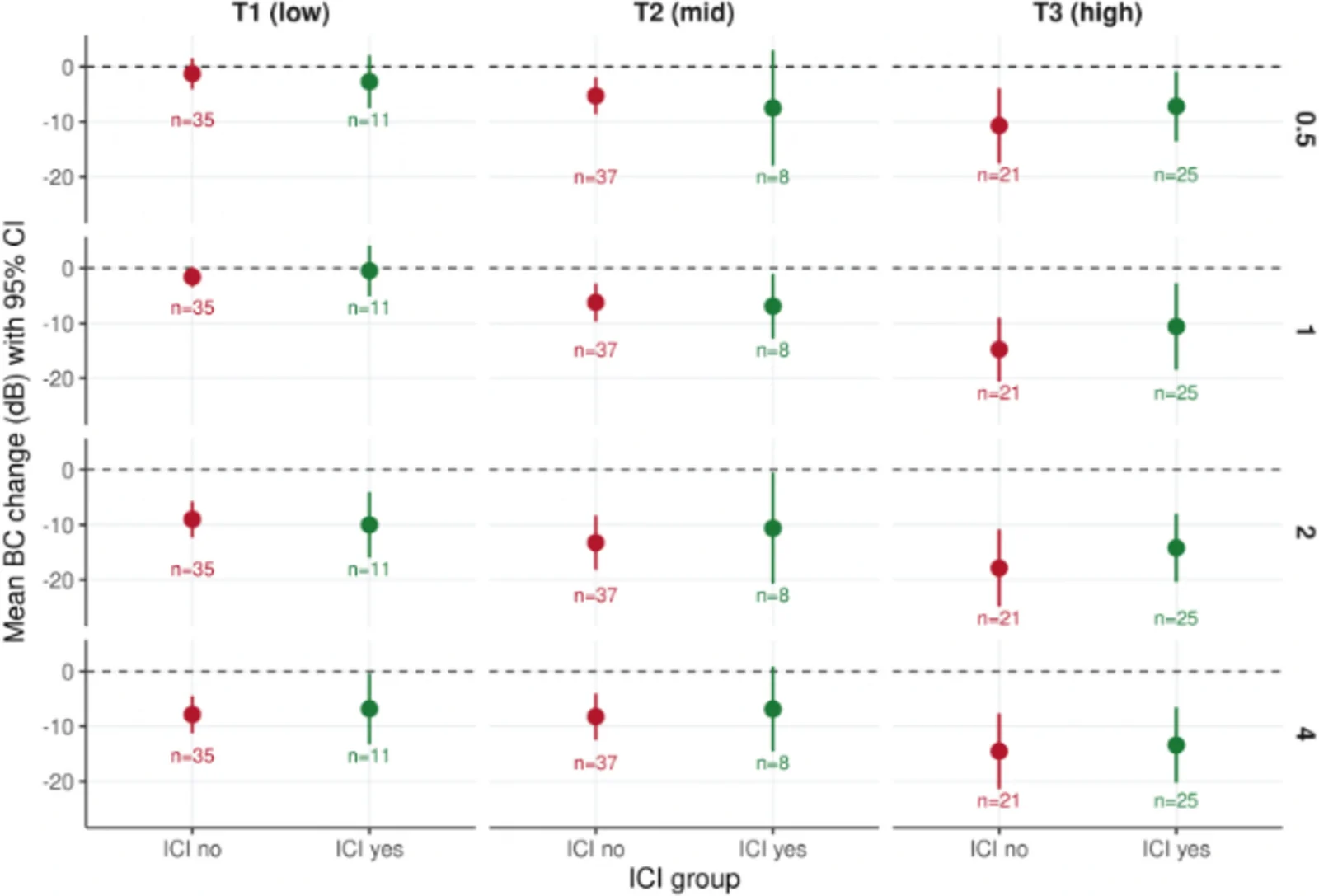
Mean BC threshold change (dB) with 95% CI by ICI group and severity tercile subgroups (unweighted)

The absolute difference in the BCPTA4 was statistically significant different from 0 (*p* = 0.008). No significant differences between the three subgroups with regard to the relative difference (*p* = 0.917) were found (Fig. 4).

In exploratory analyses by surgical modality, significant improvements in post-treatment BCPTA4 were observed after middle ear drainage by VT and LP (both *p* < 0.001). No significant improvement in post-treatment BCPTA4 was observed after middle ear drainage by PZ (*p* = 0.059). Nevertheless, given the very small sample size of the PZ group (*n* = 8), these results should be interpreted with particular caution. While there were no significant differences between the individual treatment modalities in terms of absolute or relative improvement in hearing threshold (*p* = 0.270 and *p* = 0.231 respectively), the VT group had a significantly higher number of complications (*p* < 0.001).

Across the defined surgical and tertile subgroups, no significant differences in absolute hearing threshold improvement were observed between treatment modalities (Table 3).

**Table 3:** Mean differences in ΔBCPTA4 by treatment modality in the entire cohort, in surgical subgroups and tercile subgroups with 95% CIs

Subgroup	*n*	NIC (*n*)	IC (*n*)	Mean ΔBCPTA4, NIC (dB)	Mean ΔBCPTA4, IC (dB)	Mean diff (dB)	95% CI low	95% CI high	*p *value
*Overall*	137	93	44	−8.40	−9.15	−0.75	−4.32	2.82	0.679
*Myringotomy only (PZ)*	8	6	2	−6.25	−8.75	−2.50	−18.03	13.03	0.707
*With ventilation tube (VT)*	96	72	24	−8.61	−12.50	−3.89	−8.81	1.04	0.120
*CO₂ laser myringotomy (LP)*	33	15	18	−8.25	−4.72	3.53	−1.75	8.80	0.183
*T1 (low)*	46	35	11	−4.93	−5.00	−0.07	−4.06	3.91	0.971
*T2 (mid)*	45	37	8	−8.24	−7.97	0.27	−5.75	6.29	0.927
*T3 (high)*	46	21	25	−14.46	−11.35	3.11	−4.75	10.98	0.429

## Discussion

The management of AOM primarily relies on symptomatic treatment and the targeted use of antibiotics when indicated. In cases of failure of conservative therapy or in the presence of complications, surgical intervention may become necessary [14–16]. In cases of concomitant sensorineural hearing loss, adjunctive anti-inflammatory glucocorticoids are presumed to promote recovery of toxically impaired cochlear function.

Despite the absence of standardized treatment protocols, local intratympanic glucocorticoid administration has become established in several vestibulocochlear disorders, largely due to the avoidance of systemic side effects and the achievement of higher perilymphatic drug concentrations compared to systemic administration [10–12]. However, evidence supporting the use of intratympanic glucocorticoid injections in AOM with sensorineural hearing loss remains limited [13]. The present study compares two treatment strategies for AOM with inner ear involvement: (1) therapy without intratympanic glucocorticoids (NIC) and (2) therapy including intratympanic glucocorticoids (IC).

### Subjective improvement

Overall, 65% of patients reported subjective improvement in hearing during therapy. Although the proportion was numerically higher in the NIC group (70%) compared to the IC group (55%), this difference did not reach statistical significance and should not be interpreted as evidence of clinical superiority of either regimen.

Given the comparable overall duration of therapy in both groups, differences in patient-reported outcomes are unlikely to be explained by treatment length alone. Instead, the treatment burden must be considered. While the NIC regimen involved a single invasive intervention (surgical drainage), the IC protocol required repeated intratympanic injections. Although repeated intratympanic administration after sufficient drainage is generally well tolerated, recurrent invasive procedures may constitute an additional stress factor. Psychological stress has been associated with reduced hearing recovery in patients with sudden sensorineural hearing loss, as reported by Ajduk et al. [17]. It is therefore conceivable that repeated interventions or prolonged treatment settings may negatively influence subjective perception of recovery. This potential effect should be considered when interpreting subjective outcome measures.

### Hearing threshold

A significant improvement in hearing thresholds was observed in the overall cohort during therapy (*p* < 0.001). Both the NIC and IC groups demonstrated significant within-group improvement (both *p* < 0.001), with a slightly larger effect size observed in the NIC group, confirming the general effectiveness of prompt drainage and anti-inflammatory therapy in AOM with inner ear involvement.

However, no significant difference in objective hearing gain was detected between the NIC and IC regimens. The NIC group demonstrated a numerically more consistent improvement across severity ranges and a higher proportion of clinically relevant (> 10 dB) improvements. Although these non-significant numerical trends favoured the NIC group across severity ranges they lack statistical support and may reflect baseline differences rather than true treatment effects (Figs. 2 and 4). Given baseline imbalances between groups (Table 1) and the non-randomized design, the absence of a statistically significant difference should not be interpreted as evidence of no treatment effect or as equivalence.

Three aspects are particularly relevant when interpreting these results: baseline characteristics, treatment setting, and drug delivery across the round window membrane.

Age & pre-existing conditions

Differences between groups remained regarding age, prior treatment, and baseline hearing thresholds (pre- and post-BCPTA4). Advanced age, pre-existing inner ear impairment, or more severe disease presentation may have influenced the decision to administer additional intratympanic glucocorticoids. Such confounding by indication is inherent to retrospective designs and may have attenuated potential treatment effects. IPT weighted analysis is presented in the supplement showing similar results. *Notably, the IC group was significantly older, had worse baseline hearing thresholds, and received systemic glucocorticoids less frequently, suggesting that intratympanic therapy was preferentially offered to patients with more severe or refractory disease. The absence of a detected benefit therefore does not constitute evidence of equivalence or non-inferiority, as this study was neither designed nor powered for such comparisons. Small but clinically meaningful effects of intratympanic therapy may have been obscured by residual confounding despite IPTW adjustment.*

Treatment setting

Treatment logistics differed substantially between groups. The IC regimen required repeated intratympanic applications, potentially prolonging inpatient stay or necessitating repeated outpatient visits. In contrast, after surgical drainage, the NIC protocol primarily involved systemic glucocorticoid administration, facilitating earlier discharge to a familiar environment. These differences may have influenced the overall patient experience; however, this possibility remains speculative because treatment-related stress and other contextual factors were not assessed. Although psychological stress has been associated with hearing recovery in other otologic conditions [17], the relevance of this association to patients with acute otitis media and inner-ear involvement remains uncertain.

Round window membrane

Higher perilymphatic corticosteroid concentrations after intratympanic administration have been demonstrated primarily in experimental models under non-inflammatory conditions [12]. Drug transport across the trilaminar round window membrane depends on multiple factors, including molecular properties and membrane permeability [18–22]. Whether this pharmacokinetic advantage translates into improved recovery in acute otitis media with inner-ear involvement remains uncertain, as acute inflammation may alter round-window permeability and membrane thickening in later stages may reduce drug diffusion [18–25]. Importantly, our retrospective study did not assess intralabyrinthine drug concentrations or round-window permeability. Therefore, our data do not permit conclusions regarding drug delivery across the round window and cannot explain the observed group comparison on pharmacokinetic grounds.

### Complications

The overall complication rate and need for revision surgery did not differ significantly between the NIC and IC groups (*p* = 1). However, ventilation tube (VT) placement was associated with a significantly higher complication rate compared to myringotomy with CO₂-laser (LP) or myringotomy without ventilation tube placement (PZ). Although most complications were inflammatory and self-limiting, two cases in the VT group required tympanoplasty at a later stage due to persistent tympanic membrane defects. The higher complication rate observed within VT group may partly reflect case selection and disease severity rather than the surgical technique itself, as VT was the most frequently used modality across all severity terciles (65–73%).

While no differences in hearing improvement were observed depending on the surgical technique, the higher complication rate in the VT group suggests that prolonged middle ear ventilation may not be advantageous in the setting of AOM with inner ear involvement. The primary goal of surgical intervention in the treatment of AOM with concomitant sensorineural hearing loss is the rapid reduction of inflammatory mediators. Given the typically favourable healing course after adequate therapy, long-term ventilation may expose patients to unnecessary risks, including recurrent infections or cholesteatoma formation.

Given the comparable audiometric outcomes observed in this cohort, together with the higher complication rates associated with VT placement, CO₂-laser myringotomy may warrant evaluation as an alternative approach for short-term middle-ear drainage in prospective studies. However, given the small subgroup sizes, these findings should be considered hypothesis-generating and should not be used to guide clinical practice.

Several limitations of this study should be acknowledged. The retrospective design entails an inherent risk of selection and information bias and precludes causal inference. Furthermore, treatment allocation was not randomized, introducing the potential for confounding by indication, as patients receiving intratympanic glucocorticoids may have differed in baseline severity or other clinical characteristics. This limitation was compounded by the inability to reliably reconstruct antibiotic exposure from the available medical records; consequently, differences in antibiotic regimens and treatment timing could not be accounted for, and residual confounding by antimicrobial therapy cannot be excluded. Similarly, systemic glucocorticoid therapy was not protocolized but tailored to the individual patient's clinical presentation and the treating physician's judgment, introducing an additional potential source of residual confounding. Although statistical adjustments were applied (IPTW, see supplement), residual confounding cannot be excluded. Additionally, variability in diagnostic assessment, timing of intervention, and therapeutic protocols may have influenced outcomes. Time to audiometric improvement was dependent on the timing of scheduled follow-up audiograms and should therefore be interpreted as visit-driven and interval-censored. Moreover the sample size, particularly in subgroup analyses, may limit statistical power to detect small but clinically relevant differences. Finally, as a single-center study conducted at a tertiary referral hospital, findings may not be directly extrapolable to broader patient populations, e.g. pediatric populations, outpatient-only management settings, or centers employing different diagnostic and therapeutic algorithms. Referral bias may have resulted in a disproportionate representation of more severe cases.

## Conclusion

In this retrospective non-randomized cohort, both regimens were associated with significant improvement in hearing thresholds in patients with AOM and inner ear involvement. Although non-significant numerical trends favoured the NIC group, no statistically significant differences were observed. These trends may reflect baseline imbalances rather than true treatment effects. No statistically significant between-group difference in BCPTA4 improvement was observed. Given baseline imbalances and residual confounding, this should not be interpreted as evidence of absence of an incremental effect of intratympanic glucocorticoids, nor as equivalence. Prospective controlled studies are needed before informing clinical practice.

In exploratory analyses, comparable audiometric outcomes were observed across drainage modalities, whereas ventilation tube (VT) placement was associated with higher complication rates. Given the small subgroup sizes, these findings should be regarded as hypothesis-generating and warrant prospective confirmation before informing clinical practice.

## Supplementary Information

Below is the link to the electronic supplementary material.

Supplementary file1 (DOCX 1881 KB)

## Data Availability

The datasets analysed during the current study are available from the corresponding author on reasonable request.

## Abbreviations

AOM: Acute otitis media
ATE: Average treatment effect
BCPTA4: Bone-conduction pure-tone average
Cis: Confidence intervals
GC: Glucocorticoid
HRs: Hazard ratio
IC: Intratympanic application of glucocorticoids
IPTW: Inverse probability of treatment weighting
IQR: Interquartile range
LP: Myringotomy with CO₂-laser
MCID: Minimal clinically important difference
NIC: Non-intratympanic glucocorticoid group
PZ: Myringotomy without Ventilation Tube Insertion
RWM: Round window membrane
SMDs: Standardized mean differences
TTI: Time to improvement
VT: Myringotomy with Ventilation Tube Insertion

## Declarations

The manuscript is not under simultaneous consideration with another journal, has not been published before, and all authors concur with the submission.
