# Supplementary material for: Does local steroid therapy improve outcomes in acute otitis media with serous labyrinthitis? A retrospective analysis
Source: Eur Arch Otorhinolaryngol. 2026 Oct 20;283(12):7915–28. doi: 10.1007/s00405-026-10616-y (PMC13768291; doi:10.1007/s00405-026-10616-y)
Supplement: Supplementary file 1 [file 405_2026_10616_MOESM1_ESM.docx]

**Does Local Steroid Therapy Improve Outcomes in Acute Otitis Media with Serous Labyrinthitis? A Retrospective Analysis**

**Supplementary Materials - Index**

| **Supplementary Figures and Tables** |  |
| --- | --- |
| Figures | *pag. 3-4* |
| Tables | *pag. 5* |
|  |  |
|  |  |
|  |  |
|  |  |
|  |  |
|  |  |
|  |  |
|  |  |
|  |  |
|  |  |
|  |  |

**Supplementary Figures and Tables**

**Supplementary Figures**

Propensity Score Analysis

To account for potential confounding, inverse probability of treatment weighting (IPTW) was applied based on a logistic regression model estimating the probability of receiving intratympanic glucocorticoids (ICI). The model targeted the average treatment effect and included nine baseline covariates, comprising demographic characteristics, affected ear side, surgical drainage modality, prior therapy, baseline air- and bone-conduction PTA4, as well as cohort tertiles of PTA4 and additional baseline variables as specified in the dataset.

Covariate balance between treatment groups was assessed using standardized mean differences (SMDs) before and after weighting. Continuous variables were evaluated using Hedges’ g, and binary variables using standardized differences. Absolute SMD values < 0.15 were considered indicative of acceptable balance.

Overlap of propensity scores between groups was evaluated by visual inspection of the unweighted density distributions.

**Supplementary Figure 1:** Time to improvement probability by treatment modality. IPT-weighted Kaplan–Meier. IPT-weighted Cox

**Supplementary Figure 2:** IPT-weighted mean BC threshold change (dB) with 95% CI by ICI group and severity tercile subgroups

**Supplementary Figure 3:** Absolute standardized mean differences by covariate before (Unweighted) and after PS weighting (IPTW); vertical reference at |SMD| = 0.15.

**Supplementary Tables**

| Subgroup | n | NIC (n) | IC (n) | Mean ΔBCPTA4, NIC (dB) | Mean ΔBCPTA4, IC (dB) | Mean diff (dB) | 95% CI low | 95% CI high | p value |
| --- | --- | --- | --- | --- | --- | --- | --- | --- | --- |
| *Overall* | 137.00 | 93.00 | 44.00 | -8.70 | -9.65 | -0.95 | -4.96 | 3.06 | 0.643 |
| *Myringotomy only (PZ)* | 8.00 | 6.00 | 2.00 | -6.25 | -15.97 | -9.72 | -15.65 | -3.78 | 0.018 |
| *With ventilation tube (VT)* | 96.00 | 72.00 | 24.00 | -9.17 | -10.18 | -1.01 | -6.11 | 4.09 | 0.699 |
| *CO2 laser myringotomy (LP)* | 33.00 | 15.00 | 18.00 | -7.85 | -4.66 | 3.18 | -1.63 | 8.00 | 0.205 |
| *T1 (low)* | 46.00 | 35.00 | 11.00 | -4.94 | -5.31 | -0.37 | -5.07 | 4.33 | 0.879 |
| *T2 (mid)* | 45.00 | 37.00 | 8.00 | -8.09 | -12.21 | 4.12 | -9.61 | 1.37 | 0.149 |
| *T3 (high)* | 46.00 | 21.00 | 25.00 | -13.94 | -13.43 | 0.51 | -7.72 | 8.74 | 0.905 |

**Supplementary Table 1:** IPT-weighted mean differences in ΔBCPTA4 by treatment modality in the entire cohort, in surgical subgroups and tercile subgroups with 95% Cis.

**Supplementary Table 2: Baseline air-conduction pure-tone average (ACPTA4) and air–bone gap by treatment group.**

| **Variable** | **Group** | **n** | **Median (IQR)** | **p (NIC vs IC)** |
| --- | --- | --- | --- | --- |
| Baseline BCPTA4 (analysis, dB) | Overall | 137 | 28.8 (21.2–41.2) |  |
| Baseline BCPTA4 (analysis, dB) | NIC | 93 | 28.8 (20.0–35.0) |  |
| Baseline BCPTA4 (analysis, dB) | IC | 44 | 38.8 (24.7–49.4) | 0.002 |
| Baseline ACPTA4 (dB) | Overall | 137 | 48.8 (35.0–62.5) |  |
| Baseline ACPTA4 (dB) | NIC | 93 | 45.0 (35.0–57.5) |  |
| Baseline ACPTA4 (dB) | IC | 44 | 57.5 (35.9–67.8) | 0.035 |
| Baseline air-bone gap ACPTA4-BCPTA4 (dB) | Overall | 137 | 18.8 (11.2–23.8) |  |
| Baseline air-bone gap ACPTA4-BCPTA4 (dB) | NIC | 93 | 20.0 (13.8–23.8) |  |
| Baseline air-bone gap ACPTA4-BCPTA4 (dB) | IC | 44 | 14.4 (8.8–22.8) | 0.056 |

*Values are median (1st–3rd quartile). Group comparison by Wilcoxon rank-sum test. ACPTA4 and BCPTA4 averaged over 0.5, 1, 2 and 4 kHz; air–bone gap = ACPTA4 − BCPTA4.*
